# 

**Published:** 1892-12-24

**Authors:** 


### CHRISTMAS APPEAL NUMBER.

CONTENTS.

| | PAGE |
|---|---|
| One Merry Christmas Bell | 193 |
| St. Mary's Hospital, Paddington.—The Clarence Memorial Wing | 194 |
| The Nurture of Royal Infants | 195 |
| The Doctor's Christmas | 196 |
| Christmas Thoughts at the National Gallery | 197 |
| The Christmas Numbers | 198 |
| Oxford at Its Best | 199 |
| Notes and News | 200 |
| Our Christmas Tableaux Vivants | 201 |
| Sweet Charity's Guide for Christmas Givers (Being the Christmas Number of THE HOSPITAL) | 203 |
| GENERAL HOSPITALS | 203 |
| SPECIAL HOSPITALS. — Consumption, 206; Epilepsy and Paralysis—Children—Lying-in, 207; Women—Miscellaneous Special | 208 |
| A FEW OTHER CHARITIES | 209 |
| Where to Send Christmas Boxes | 210 |
| Miss Ethel M. Warner and Mr. Lawson Tait | 210 |

EXTRA SUPPLEMENT.—THE NURSING MIRROR.

| | |
|---|---|
| En Passant.—A Happy Christmas—Toronto General Hospital—Cumberland Infirmary—An Apology—St. Lawrence's Catholic Home—Nursing Scheme at Gateshead—"Fuss"—Short Items | xcv |
| Where Some of our Nurses are Spending Christmas—Nursing in the Land of Ophir | xcvi |
| Christmas to an Invalid.—The Kingdom of God is Within You | xcviii |
| Christmas in Hospital.—A Nurse's View | xcviii |
| Help the Nurses to Help the Sick | xcix |
| Appointments | c |
| Poor Barker | c |

